# NEDD8, stress granules, and amyotrophic lateral sclerosis: unveiling the therapeutic potential of the NEDP1 protease

**DOI:** 10.1042/EBC20253036

**Published:** 2025-12-12

**Authors:** Dimitra Mitsiadou, Dimitris P. Xirodimas, Jolanta Polanowska

**Affiliations:** 1 CRBM, Univ. Montpellier, CNRS, Montpellier, France

**Keywords:** amyotrophic lateral sclerosis, NEDD8, NEDP1/SENP8, PARP1, stress granules

## Abstract

Protein quality control (PQC) systems are crucial for maintaining cellular proteostasis, particularly under stress that promotes misfolded protein accumulation. A central component of this response is the assembly of stress granules (SGs), cytoplasmic condensates of RNA and proteins that temporarily stall translation. Aberrant SG dynamics, often linked to mutations in SG proteins, contribute to neurodegenerative diseases such as amyotrophic lateral sclerosis (ALS), where persistent protein aggregates are hallmarks. This review examines the emerging role of the ubiquitin-like modifier NEDD8 and its deconjugating enzyme NEDP1 in regulating SG homeostasis. Recent studies identify NEDP1 as a critical factor controlling SG clearance. Inhibition of NEDP1 enhances SG turnover, prevents pathological solidification, and promotes the disassembly of toxic aggregates through hyper-NEDDylation of PARP1, a DNA repair enzyme that also governs SG dynamics. Unlike broad-spectrum PARP1 inhibitors, which can impair DNA repair and cause cytotoxicity, NEDP1 inhibition offers a stress-specific approach that preserves normal cellular functions. Encouragingly, NEDP1 inhibition effectively causes aggregate elimination in ALS patient-derived fibroblasts and restores motility in *Caenorhabditis elegans* disease models. Altogether, these findings highlight NEDP1 as a key regulator of SG regulation and a promising therapeutic target for ALS and related neurodegenerative disorders.

## The protein quality control and management of stress-induced protein misfolding

Mammalian cells depend on their protein quality control (PQC) systems to manage and mitigate the negative impacts of misfolded proteins [1,2]. PQC supports correct protein folding, prevents aggregation, and eliminates irreparably damaged proteins through the ubiquitin–proteasome system (UPS) and autophagy [1,3–5]. The UPS removes defective proteins and also regulates normal cellular processes such as gene expression and signaling [6]. Autophagy complements UPS activity, particularly during proteotoxic stress, by directing protein aggregates to lysosomes [7].

Environmental or pathological stress can trigger misfolded protein buildup, disrupting normal cellular functions. In response, eukaryotic cells activate stress signaling, reorganize nucleocytoplasmic transport, suppress global translation, and enhance chaperone expression to restore protein integrity [8–12]. A hallmark of this stress adaptation is the formation of stress granules (SGs)—membrane-less assemblies of stalled mRNAs and RNA-binding proteins (RBPs) [13,14]. SGs help restore cellular balance and influence cell survival outcomes [8,15]. PQC factors regulate SG dynamics, maintaining their liquid-like state while processing misfolded proteins; failure to do so may lead to harmful aggregates, linked to neurodegenerative disorders [15,16].

Although SGs are typically cytoplasmic, recent evidence indicates that similar assemblies known as nuclear stress bodies can form within the nucleus in response to DNA damage, viral infection, or chemotherapy [17]. This is particularly significant as the nuclear proteome is enriched in disordered proteins, making it susceptible to proteotoxic stress. Cells appear to compartmentalize aberrant proteins within such assemblies, and current findings reveal distinct PQC pathways in the cytoplasm and nucleus. These involve specialized chaperones, E3 ligases, long non-coding RNAs, and compartment-specific proteasomes to meet localized proteostasis demands [18–23].

## SGs in physiology: assembly and elimination

SGs are dynamic membrane-less cytoplasmic condensates that assemble in response to various stresses, such as oxidative stress, heat shock, or endoplasmic reticulum (ER) stress through liquid–liquid phase separation (LLPS). They are composed of translation initiation factors (e.g., eIF2α, eIF3), RBPs (e.g., G3BP1/2, TDP-43, TIA-1, TIAR), and stalled mRNAs. The formation of SGs is considered as part of a protective mechanism which temporarily halts protein synthesis, preserves the translation machinery and RNAs, while allowing cells to prioritize stress response pathways over housekeeping processes. Once stress is relieved, the granules disassemble, liberating their components and allowing them to resume their normal functions [24,25].

Several posttranslational modifications (PTMs) modulate SG dynamics by influencing protein interactions, subcellular localization, stability, and phase separation properties [26–30]. A key event in SG formation is the phosphorylation of eIF2α by one of the Integrated Stress Response kinases (HRI, PERK, PKR, or GCN2), which restricts active eIF2-GTP/Met-tRNAi ternary complex availability [31–33].

Other PTMs, including mono- and poly-ADP ribosylation (MARylation and PARylation, respectively), are mediated by ADP-ribosyltransferases (ARTs, also known as PARPs [34]), notably PARP1 but also PARP12 and PARP13, which are central regulators of SG assembly and composition. PARP1, primarily nuclear, generates poly(ADP-ribose) (PAR) during stress which acts as a molecular signal, recruiting proteins like PARP12 from the Golgi to SGs in the cytoplasm [35–38]. This PARP1-driven process is reversible and crucial for maintaining Golgi function and protein trafficking; inhibition or depletion of PARP1 prevents PARP12’s SG localization and partially restores Golgi integrity. PARP13, in contrast, regulates SG dynamics by binding PAR, functioning as a molecular hub with the dual capacity to interact with both RNA and PAR. These interactions promote SG assembly and fusion events, thereby controlling the overall SG size and number [39]. Together, these findings establish that PARP1-generated PAR chains orchestrate the recruitment and function of PARP12 and PARP13 in SGs. Additionally, recent studies have highlighted PARP10 as a pivotal enzyme for the initiation of SG assembly, highlighting PARP10-driven MARylation of G3BP1 as a critical step for SG nucleation. Knockdown of PARP10 markedly impairs G3BP1-mediated SG assembly and alters the SG core by reducing translation factor content [40]. Together, these findings emphasize that the co-ordinated actions of multiple PARPs provide a finely tuned regulatory network that ensures proper SG dynamics, cellular stress adaptation, and, through ADP-ribose–mediated docking interactions, promotes scaffold formation and protein recruitment within SGs [35–37].

RNAs are key components of SGs and defining their role in dynamics has been an intense area of research [41–44]. While long non-coding and intergenic non-coding RNAs are present, mRNAs dominate (≈89.5%), due to the tight connection of SGs and translation [41,43]. Importantly, while SG-associated transcripts represent only ~10% of the total cellular mRNA pool, they are enriched relative to proteins within granules, reflecting the central role of RNAs in driving condensate assembly [43]. By engaging in RNA–RNA and RNA–protein interactions, RNAs drive assembly and control the LLPS of RBPs with sequence and length, and are actively remodeled by RNA helicases such as DDX3X, which facilitate turnover of RNA–protein complexes [45]. Understanding the role of RNAs inside the granules can aid in further deciphering the granule code and revealing therapeutic targets for diseases linked to dysregulated SG dynamics.

SG clearance is a highly regulated and context-dependent process, distinct from the degradation of misfolded proteins. Unlike aggregated proteins marked for destruction, SGs are typically remodeled upon resolution of stress rather than being simply degraded [46]. SG disassembly often coincides with translational reactivation and is modulated by the type and duration of the stressor. Acute stressors, such as heat shock or oxidative stress, generally induce transient SGs that are efficiently cleared through chaperone-mediated dissolution. This mechanism is considered a primary route of SG resolution. In contrast, chronic stress leads to the formation of more persistent SGs (alternatively mentioned as aberrant in literature [46,47]), often containing misfolded proteins, which require selective autophagy—referred to as granulophagy—for clearance. SG disassembly proceeds through a stepwise process involving the release of RNAs and proteins from the granule core. Molecular chaperones, including HSP70, BAG3, HSPB8, and VCP/p97, mediate ATP-dependent remodeling and facilitate the degradation of misfolded components [48–50].

Finally, PTMs such as ubiquitination and SUMOylation critically shape SG composition and fate. The UPS regulates SG dynamics, with proteasome subunits and ubiquitin conjugates enriched in SGs under heat stress [51,52]. K48-linked ubiquitin at SG peripheries is degraded by 26S proteasomes, with USP5 and USP13 facilitating clearance after heat stress, whereas under arsenite exposure ZFAND1 recruits proteasomes instead [53–55]. These stress-specific differences likely reflect distinct PQC substrates, including misfolded proteins and defective ribosomal products [46,49,53]. The adaptor UBQLN2 further restricts SG assembly and promotes clearance via ubiquitin-dependent phase separation [56,57]. SUMOylation also protects against TDP-43 aggregation within SGs and, through RNF4-mediated SUMO-primed ubiquitination of RBPs, drives SG clearance after stress, linking nuclear and cytoplasmic quality control pathways and depending on stress-induced inhibition of SUMO deconjugases [58–61].

Granulophagy is vital in SG clearance, particularly under chronic stress. Autophagy-related proteins, including the ATG1-ATG13 kinase complex, localize to SGs during heat-induced stress and redistribute following SG elimination [62,63]. Inhibition or knockdown of core autophagy components such as ATG3, ATG5, or ATG7 results in SG accumulation, whereas pharmacological activation of autophagy (e.g., with rapamycin) accelerates SG clearance [64,65].

## Defective SG elimination and pathological consequences

Impaired SG clearance has been linked to several neurodegenerative disorders, including amyotrophic lateral sclerosis (ALS), frontotemporal dementia (FTD), Alzheimer’s, and Parkinson’s disease. Mutations in SG-associated proteins, such as TDP-43, FUS, and VCP, interfere with phase separation or nucleocytoplasmic transport, leading to persistent SGs that sequester essential proteins and RNAs. Additionally, chronic stress exposure, or failures in cellular clearance mechanisms can result in aberrant SGs. These persistent granules disrupt translation and cellular homeostasis, ultimately contributing to disease pathology [50,66]. Such impairments may drive an irreversible liquid-to-solid phase transition within SGs, especially when coupled with disease-linked PTMs and mutations in prion-like domains or intrinsically disordered regions of SG proteins [67,68]. These aggregates interfere with key cellular processes, including RNA metabolism, stress signaling, and translational re-initiation, thereby contributing to cellular dysfunction and death [24,69–74]. It has been suggested that SGs act as a cellular defense mechanism that can mitigate neurodegenerative cascades by sequestering toxic proteins and managing cellular stress responses, rather than contributing to neurodegeneration pathology directly [66,75,76]. However, persistent SGs are strongly associated with neurodegenerative diseases, although the exact role still remains unclear—are SGs the initiators of the pathology, or is their accumulation simply a consequence of pre-existing proteostatic failure? And critically, would clearance of aberrant aggregates ameliorate ALS-related defects?

Among neurodegenerative diseases, ALS has the strongest association with SG pathology. A hallmark of ALS is the cytoplasmic accumulation of TDP-43 aggregates, which often but not always colocalize with SGs in patient spinal cord tissue. Upon cellular stress, TDP-43 translocates from the nucleus to the cytoplasm, where it modulates SG dynamics. Notably, TDP-43 aggregates colocalize with SGs in spinal cord tissue from ALS patients [77]. While TDP-43 can aggregate independently of SGs, optogenetic studies have demonstrated that chronic SG induction is sufficient to drive TDP-43 aggregation, suggesting that pathological SG persistence may actively contribute to disease progression [59,78,79]. TDP-43 also regulates G3BP1 mRNA stability, and its nuclear depletion or ALS-linked mutations reduce G3BP1 levels, weakening the SG response [80].

Other ALS-associated proteins similarly contribute to pathological SG behavior, among others we can cite FUS, SOD1, and profilin-1. Recent work has shown that mutant FUS reshapes the SG transcriptome, switching to a more unstructured, AU-rich RNA composition that prolongs SG persistence, while mutant SOD1 aberrantly interacts with G3BP1 in an RNA-independent manner [81,82]. Importantly, mutant SOD1 inclusions colocalize with G3BP1-positive granules in spinal cord motor neurons from ALS mouse models, with nearly all motor neurons containing SOD1 inclusions also harboring G3BP1 co-inclusions. Profilin-1, another SG-associated protein, shows impaired assembly, targeting, or clearance in ALS-linked mutants, and mutant profilin-1 aggregates can induce prion-like TDP-43 conformations, amplifying SG-related toxicity [83,84].

Genetic risk factors such as intermediate ATXN2 polyQ expansions, VCP and HNRNPA1/A2B1 mutations, and defects in nucleocytoplasmic transport or autophagy further impair SG clearance, sensitizing motor neurons to aggregation [85–89]. Notably, hexanucleotide repeat expansions in C9orf72—the most common genetic cause of ALS and FTD—decrease C9orf72 protein levels, resulting in defective autophagy-mediated elimination of SG via the p62 pathway [90]. Evidence from patient tissue, iPSC-derived motor neurons, and animal models links maladaptive SG behavior to impaired proteostasis, RNA dysregulation, and axonal degeneration, while biochemical studies show that PTMs such as G3BP1 phosphorylation, RGG methylation, and HSP70/HSPB8–BAG3 chaperone activity modulate SG material properties and reversibility [46,49,91].

The complex interactions between SGs, TDP-43, and the rest of the ALS-associated proteins underscore the need to unravel the precise mechanisms driving SG aggregation and their contribution to ALS progression, which could pave the way for therapeutic strategies aimed at restoring SG dynamics or preventing toxic aggregate formation.

## The NEDD8 pathway

Neural precursor cell expressed developmentally down-regulated protein 8 (NEDD8) is a member of the ubiquitin-like protein family, sharing about 60% sequence identity with ubiquitin. It is essential in most eukaryotes, except *Saccharomyces cerevisiae* [92–94]. Like ubiquitin, NEDD8 modifies proteins through a three-enzyme cascade (E1, E2, E3), forming an isopeptide bond with lysine residues on target proteins. Prior to its activation, NEDD8 is processed by proteases to expose a C-terminal di-glycine motif [94]. Normally, NEDD8 is conjugated to substrates through its dedicated enzymatic cascade—E1 NAE (APP-BP1/Uba3), E2s UBE2M or UBE2F, and a set of NEDD8-specific E3 ligases—constituting canonical NEDDylation [95,96]. Under stress conditions such as heat shock, however, NEDD8 can also be activated and transferred by components of the ubiquitin pathway in an alternative route termed atypical NEDDylation. Atypical NEDDylation has been linked to the handling of protein aggregates in both cytoplasm and nucleus, where it can modulate properties of condensates [20,95–97]. Atypical NEDDylation co-opts ubiquitin E1/E2 enzymes and is likely to be differentially sensitive to pharmacological NEDD8 pathway inhibitors. These mechanisms and their implications for proteostasis and condensate biology are comprehensively reviewed by Meszka et al. [96].

**Figure 1:**
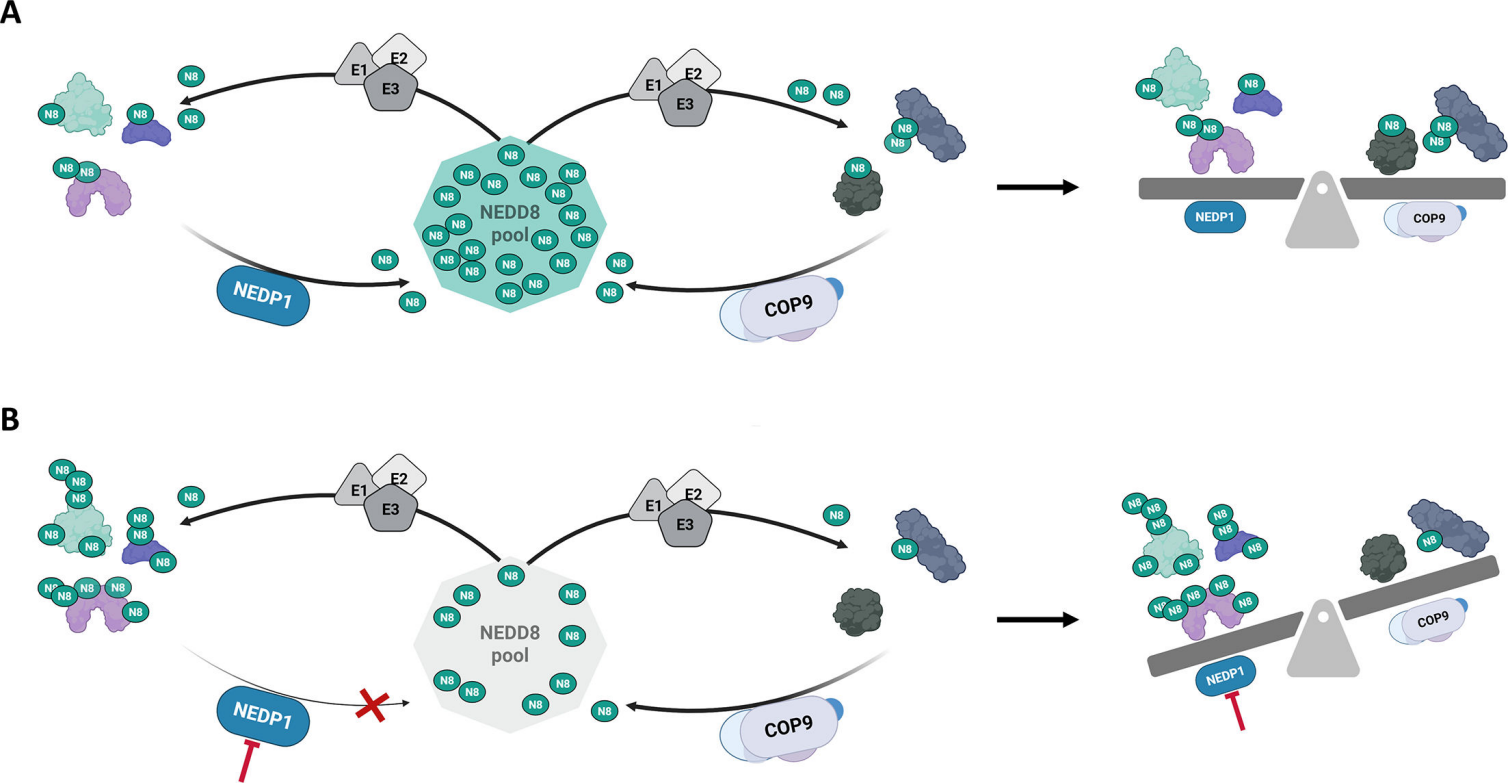
The NEDD8 cycle. **A**. Conjugating (**E1, E2, E3**) and deconjugating enzymes (COP9, NEDP1) provide the equilibrium for protein NEDDylation. **B**. Inhibition of the deconjugating enzyme NEDP1 disrupts the equilibrium resulting in NEDD8 accumulation on NEDP1-dependent NEDD8 substrates, depletion of the pool of free NEDD8, and subsequent decrease in the NEDDylation of the COP9-dependent NEDD8 substrates (mainly cullins).

NEDDylation is a reversible process (Figure 1A). The metalloprotease COP9 signalosome (CSN) and the cysteine protease NEDP1 (also called SENP8 or DEN1) remove NEDD8 from proteins [98–100]. NEDP1 is also the main enzyme that processes the inactive NEDD8 precursor but can be replaced by enzymes like UCH-L3. CSN strictly deconjugates NEDD8 from cullins—scaffold proteins in cullin-RING ligases (CRLs), which are major E3 ubiquitin ligase complexes. NEDDylation activates CRLs, whereas deNEDDylation by CSN regulates their activity [101]. In contrast, the broader role of NEDP1 in protein regulation remains less characterized.

## NEDP1-dependent NEDD8 substrates

Numerous NEDP1-dependent NEDD8 substrates have been identified individually across various organisms, laying the foundation for large-scale efforts to define the NEDDylated proteome [102–109]. In the past decade, several groups have leveraged mass spectrometry-based proteomics to systematically identify substrates regulated by NEDP1 [97,110–113]. These studies employed the use of antibodies that specifically recognize the diglycine (K-εGG) remnant (diGly) on lysine residues that remain on NEDD8 modified peptides following trypsin digestion [110,114]. Quantitation of the abundance of diGly signatures upon NEDP1 deletion allowed the identification of potential NEDP1-dependent NEDD8 substrates. However, as ubiquitin and ISG15 also provide diGly remnants upon trypsin digestion, a key limitation of this approach was its inability to differentiate between NEDD8, ubiquitin, or ISG15 modifications.

**Figure 2:**
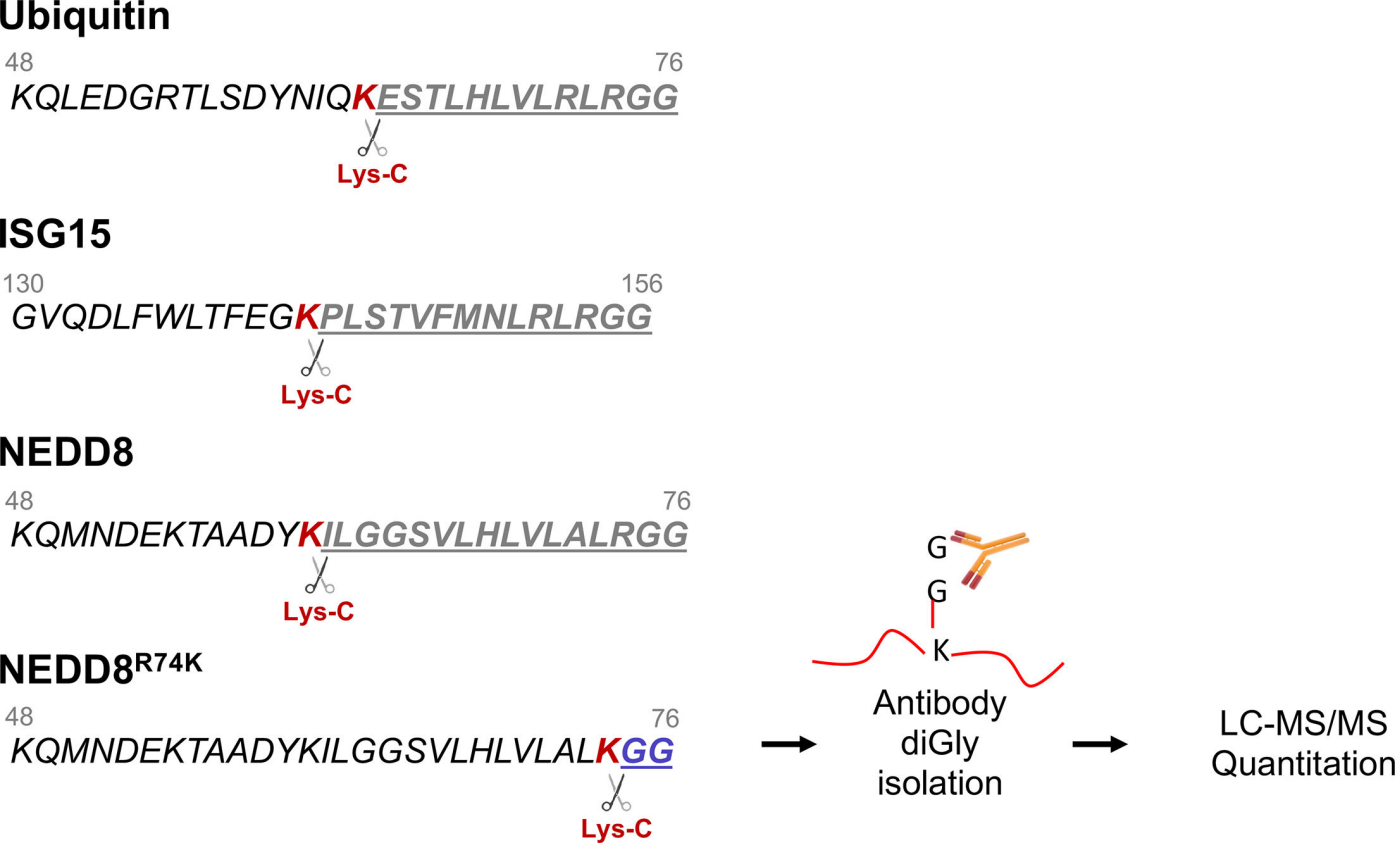
The use of the NEDD8^R74K^ mutant allows the generation of –GG signatures on peptides upon Lys-C digestion specifically from NEDDylated^R74K^ proteins.

To overcome this issue, Vogl et al. and Lobato-Gil et al. independently developed a method for specifically identifying NEDD8-modified lysines. They combined the use of a NEDD8 mutant (NEDD8^R74K) with Lys-C endoproteinase sample digestion instead of the commonly used trypsin that cleaves both after Lysine and Arginine residues. That ensured the generation of peptides with diGly remnants only from substrate proteins modified with the NEDD8^R74K mutant but not with ubiquitin, ISG15, or endogenous NEDD8 (Figure 2) [97,111]. Although validation experiments confirmed that NEDD8^R74K retained functional activity, concerns remained that the R74K mutation, located near the C-terminus, could affect substrate recognition or processing efficiency, potentially limiting the sensitivity of the proteomic output.

Further refining the methodology, Kassouf et al. capitalized on the observation that elevated NEDDylation in NEDP1-deficient (KO) cells is mediated exclusively by the canonical NEDD8 conjugation machinery and it is not affected upon inhibition of the ubiquitin system that blocks atypical NEDDylation (Figure 1B) [115]. To reduce the levels of diGly signatures derived from ubiquitin modification, they treated cells with the ubiquitin E1 inhibitor MLN7243. This treatment effectively suppressed ubiquitin-derived K-εGG peptides with no effect on NEDD8 modification, therefore enabling a more robust quantitation of NEDP1-dependent NEDDylation events and a more accurate mapping of the NEDD8-modified proteome under conditions of endogenous wild type NEDD8 expression [112].

The high number of NEDP1-dependent NEDD8 substrates that have been identified through all the studies mentioned above highlights the important role of NEDP1 in various pathways, through NEDDylation control [115]. However, the regulation of NEDP1 activity remains poorly understood with limited and fragmented information available across various organisms. Long et al. reported that NEDP1 splicing is modulated by the long non-coding RNA MALAT1, in small intestine and colon tissues of a mouse intestine tumorigenesis model [116]. Additionally, NEDP1 is subject to PTMs such as ubiquitination and phosphorylation, observed in *Arabidopsis thaliana* and *Aspergillus nidulans,* respectively [113,117].

Our current knowledge of NEDP1 regulation and function largely stems from studies on its role in the DNA damage response. NEDP1 protein levels rise under various stress conditions, such as UV exposure and chemotherapeutic treatment, through post-transcriptional mechanisms that promote apoptosis. By limiting poly-NEDD8 chain formation, NEDP1 enhances mono-NEDD8 accumulation, which signals apoptosome formation via HSP70 ATPase activation. Under oxidative DNA damage, NEDP1 also reduces NEDD8 modification of PCNA and/or the RAD18 E3 ligase, thereby facilitating PCNA–Polη interactions, a key step in the translesion DNA synthesis pathway [115,118].

## NEDP1 as a master regulator of SG dynamics

Recent work has uncovered a central role for the Ubl modifier NEDD8—specifically its removal by the deNEDDylating enzyme NEDP1—in regulating SG dynamics. Although ubiquitin and SUMO are well-established in SG biology, NEDD8’s function had been unclear. Jayabalan et al. reported that long-term inhibition of the NEDD8 pathway impairs arsenite-induced SG formation and that multiple SG-resident RBPs, including SRSF3, are NEDD8 substrates [119]. In contrast, Markmiller et al. observed no effect on SGs after short-term NEDDylation inhibition—likely reflecting selective inactivation of CRLs without perturbing the broader, non-cullin NEDDylation that influences SG material properties [120]. Together, these findings support a context-dependent model: RBP NEDDylation promotes rapid SG nucleation during acute stress, whereas proper deNEDDylation by NEDP1 is required for SG remodeling and clearance during recovery.

This gap was directly addressed by Kassouf *et al*., who identified NEDP1 as a key modulator of SG turnover [112]. Under both arsenite and heat shock, NEDP1 deletion accelerated SG assembly and enhanced SG clearance. These alterations occurred without changes in core SG protein levels, implicating elevated NEDDylation as the causal factor. Inhibition of NEDD8-activating enzyme (NAE) with MLN4924 reversed the SG phenotype in NEDP1-deficient cells, directly linking NEDD8 signaling to SG regulation. Using a nanobody specifically targeting NEDP1, the team transiently suppressed NEDP1 function, reproducing the phenotypes seen in cells genetically devoid of NEDP1.

The accelerated clearance of SGs in NEDP1-inhibited conditions was directly linked to the biophysical properties of SGs, as fluorescence recovery after photobleaching (FRAP) experiments revealed that NEDP1-deficient cells prevent the transition of SGs from a liquid-liquid to a solid phase that is observed in control cells upon prolonged stress. The above phenotypes upon NEDP1 inhibition were linked to enhanced survival under arsenite stress, suggesting a protective effect of the increased turnover for SGs upon NEDP1 inhibition.

To decipher the mechanism behind these phenotypes, proteomic analysis using diGly-enrichment and selective ubiquitin inhibition (UBA inhibitors) was conducted as already mentioned above [112]. This approach enabled the identification of over 900 NEDD8-conjugated proteins under native conditions, many of which were linked to SGs—including G3BP1, translation initiation factors (eIF4A1, eIF3D, eIF3E), and RBPs like HNRNPA1 and HNRNPK [112]. These findings suggested that NEDP1 regulates SG composition and turnover by directly controlling the NEDDylation state of key SG constituents.

Among the newly identified substrates, PARP1 emerged as a particularly compelling target. Known for promoting SG formation via PARylation, PARP1 was previously identified as a NEDD8 substrate in proteomic studies by Vogl et al. and Lobato-Gil et al. [97,111]. Additionally, previous studies indicated that PARP1 activity is controlled upon non-covalent interaction with unanchored NEDD8 trimers [121]. Kassouf et al. showed that hyper-NEDDylation of PARP1 upon NEDP1 inhibition compromises PARP1 activation during oxidative stress [112]. Critically, in an epistasis-like experiment, inhibiting PARP1 with Olaparib in control cells produced SG phenotypes observed in NEDP1 deficient cells (smaller, rapidly disassembling SGs). The same experiment had no additional effect in NEDP1 knockout cells. This suggests that NEDP1 and PARP1 are functionally linked to control SG dynamics. This notion is further strengthened with the use of PARP1 NEDDylation-deficient mutants that rescued all phenotypes observed in NEDP1 knockout cells. Specifically, compared with wildtype PARP1, PARP1 mutants produced elevated PAR levels upon oxidative stress and generated large and persistent SGs. This is also consistent with the concept that increased levels of PARP1 activity and PAR production, which is often observed in neurodegenerative diseases such as ALS, can generate large and often aberrant SGs. Interestingly, NEDP1’s role in regulating PARP1 activity appears stress specific, as its inhibition does not significantly alter responses to camptothecin, etoposide, or UV-C, suggesting that its regulatory effects depend on the type of DNA damage [112].

Based on these findings, a critical issue was to determine the effect of NEDP1 in the clearance of pathological aberrant aggregates related to SGs. In several *in vitro* model systems, including mouse neurons (TIA-1 mutations) and fibroblasts derived from patients with sporadic ALS mutations, the authors showed that NEDP1 inhibition (delivery of NEDP1 nanobodies) accelerated the elimination of ALS-related aggregates [112]. These promising results were further evaluated *in vivo* using *Caenorhabditis elegans* as a model organism, that has emerged as an attractive model system for neurodegenerative diseases including ALS. Exposure of transgenic animals with common ALS genetic defects, such as the SOD1G85R mutation or the genetic expansion within the first intron of the C9orf72 gene to oxidative stress, displays key ALS-related phenotypes: the aberrant aggregates related to SGs in motor neurons, accompanied by locomotor defects in the animals, that arise secondary to muscle dysfunction. Strikingly, deletion of ULP-3, the NEDP1 homolog in nematodes, reversed both phenotypes. In the SOD1G85R model, ULP-3 loss produced a complete rescue of motility, while a partial rescue was observed in the C9orf72 animals, most likely due to the diverse and multiple mechanisms through which the most common genetic cause of ALS induces pathology. More broadly, the observation that NEDP1 inhibition ameliorates ALS-relevant phenotypes driven by distinct genetic etiologies (e.g., SOD1G85R and C9orf72) argues for a central, mutation-independent role of NEDP1 in the elimination of pathological aggregates and in the correction of associated functional deficits. The studies in *C. elegans* illustrated that the improvement in ALS-like outcomes upon NEDP1 loss tightly correlates with clearance of neuronal aggregates, reinforcing the view that NEDP1 is a pivotal regulator of SG dynamics and proteostasis. Collectively, these data nominate NEDP1 as a potential therapeutic candidate for neurodegenerative diseases characterized by persistent granules and proteostatic collapse; however, while the worm studies are compelling, rigorous validation in mammalian disease models and patient-relevant systems will be essential before translational applications can be credibly pursued.

## PARP1 and the SG axis in ALS therapeutics

Therapies that target SGs have become a key focus in ALS research, as these cellular structures play a major role in protein clumping and nerve cell damage. To explore this, Uechi et al. screened 1600 small molecules from the Pharmakon library using HeLa cells that express GFP-tagged FUS to monitor SG formation under stress [122]. Among the compounds tested, lipoamide stood out for its ability to quickly break down existing SGs, helping restore the proper location of ALS-related proteins in the nucleus and reducing disease-related effects.

Targeting SGs through autophagy has also shown significant therapeutic promise in ALS. Pharmacological activation of autophagy using compounds like rapamycin or trehalose has been shown to promote SG clearance and reduce insoluble TDP-43 aggregates in mouse models and ALS patient-derived neurons [123–125]. Mechanistically, TRIM21 has been identified as an important factor in SG regulation; its knockdown enhances SG formation, while autophagy receptors—localized near SGs—interact with the SG protein G3BP1 under stress conditions, linking autophagy directly to SG degradation [126]. These findings support a working model in which TRIM21 and autophagy machinery co-operate to maintain SG homeostasis.

In addition to autophagy, enhancing cellular proteostasis through the proteasome system offers another promising strategy. The transcription factor NRF1 (encoded by NFE2L1) regulates the expression of proteasome subunits and is critical for maintaining PQC. Sedlacek et al. identified a series of small molecules, including bis(phenylmethylen)cycloalkanone derivatives, that activate NRF1-dependent pathways [127]. These compounds stimulate proteasome synthesis, heat shock responses, and autophagy in both cell cultures and *C. elegans* models. Notably, they reduce protein aggregate size and number without inhibiting the UPS.

Other therapeutic strategies target SG formation directly. Small molecules like ISRIB and PERK inhibitors (e.g., GSK2606414) act by modulating eIF2α phosphorylation, thus suppressing SG assembly during cellular stress [128]. In parallel, phase separation inhibitors and designer peptides targeting low-complexity domains disrupt the abnormal aggregation of key ALS proteins such as FUS and TDP-43 [129,130]. These tools address the core biophysical mechanisms driving SG pathology.

Antisense oligonucleotides (ASOs) have emerged as one of the most advanced therapeutic strategies for ALS, particularly in genetically defined subtypes such as *SOD1*- and *C9orf72*-associated ALS. Building on preclinical success, ASOs demonstrated robust efficacy in animal models by lowering toxic RNA or protein species, reducing SG burden, and restoring neuronal function. These promising findings translated into clinical development, most notably with tofersen, an ASO targeting *SOD1* mutations. In a phase III trial, tofersen reduced cerebrospinal fluid (CSF) *SOD1* protein levels and plasma neurofilament light chain, a biomarker of axonal injury, providing strong evidence of target engagement and biological activity [131,132]. For *C9orf72*-associated ALS (c9ALS), ASOs such as BIIB078 were designed to reduce toxic sense RNA foci and dipeptide repeat proteins. While preclinical studies supported their therapeutic potential, the phase I trial of BIIB078 failed to demonstrate clinical efficacy. Despite broad distribution throughout the CNS, BIIB078 did not in fact adequately suppress G4C2-related pathobiology in tissue [133,134]. Nevertheless, ongoing refinement of ASO chemistry, delivery methods, and patient stratification strategies continues to support their potential as personalized therapies for ALS and related neurodegenerative disorders. Additionally, pharmacologic chaperones like arimoclomol—an inducer of heat shock proteins such as HSP70—support protein folding and facilitate SG disaggregation, enhancing motor neuron viability [135].

At the same time, attention has also turned to the role of PARP1 and its modification product, PAR, in ALS. Blocking PARP1 activity—through either genetic methods or drugs—has been shown to reduce nerve cell damage in several ALS models, including human stem cell–derived neurons and *C. elegans*. PAR has been found to encourage the harmful clustering of proteins like FUS, TDP-43, and hnRNP A1 through phase separation, but this can be counteracted with PARP1 inhibitors [136,137]. Alirzayeva et al. further discovered that PARP1 and histone H1.2 interact abnormally with a mutant form of FUS (P525L) in ALS [138]. Reducing PARylation or the levels of H1.2 helped decrease toxic protein buildup and cell damage in both human and worm models. These findings suggest that abnormal PARylation and changes in chromatin structure contribute to ALS. Importantly, some PARP1 inhibitors, like veliparib, are already in advanced clinical use and have shown protective effects in other neurological diseases [139]. Additionally, PAR activity increases when it interacts with mutant huntingtin (HTT), linking it to other conditions like Alzheimer’s, Parkinson’s, and cerebellar ataxia [140]. Overall, these studies support the idea that controlling PARP1 activity and SG behavior could be an effective strategy for treating ALS and other neurodegenerative diseases.

## NEDP1 as a therapeutic target on neurodegeneration: targeting NEDP1 over PARP1

NEDDylation has emerged as a critical PTM in neuronal development and synaptic regulation, with multiple studies demonstrating its essential role in synaptic transmission and neuronal maturation. For instance, NEDDylation is required for presynaptic clustering of mGlu7 and the maturation of presynaptic terminals, while the deNEDDylating enzyme NEDP1 has been shown to regulate neuronal development [141,142]. Inhibition of this pathway disrupts spine formation, destabilizes synapses, and impairs cognitive function, underscoring its importance for neuronal connectivity and plasticity [143]. A recent review further emphasized how both normal and abnormal NEDDylation influence central nervous system physiology and disease development [144]. Extending these insights, the ablation of NEDD8 in young glutamatergic neurons was shown to alter the expression of developmental transcription factors, thereby preventing the establishment of a mature glutamatergic neuronal phenotype [145]. Together, these findings establish NEDDylation as a fundamental regulator of neuronal differentiation, synaptic stability, and higher-order brain functions.

**Figure 3:**
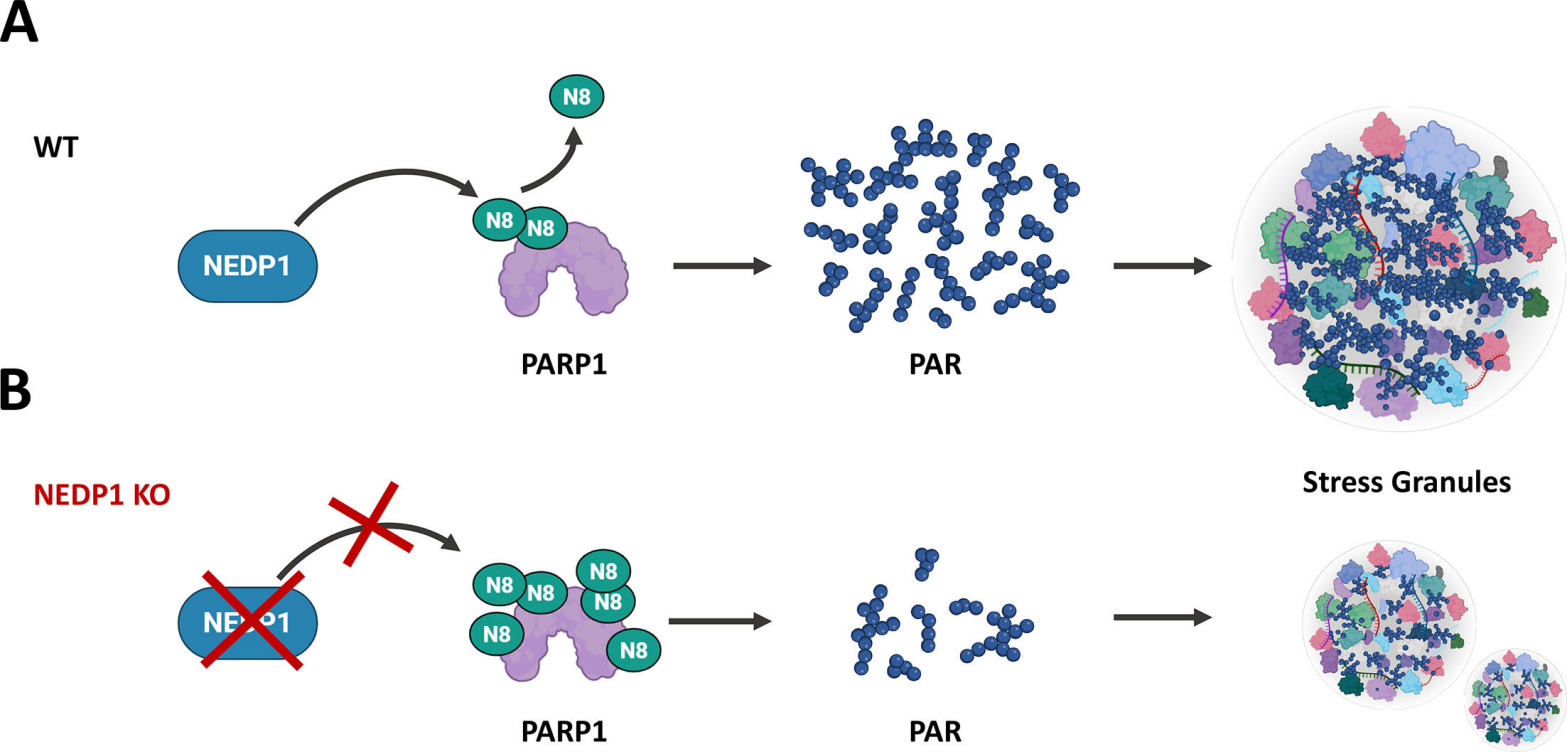
NEDP1 ‘buffers’ PARP1 activity during stress for the formation of SGs. **A.** NEDP1 controls the level of PARP1 NEDDylation and subsequently the production of the required poly-ADP-ribose (PAR) during oxidative stress for the generation of SGs. **B.** Inhibition of the NEDD8 deconjugating enzyme NEDP1, causes the hyper-NEDDylation of PARP1, resulting in decreased PARP1 activity during oxidative stress, evident by reduced levels of PAR. The low levels of PAR result in the generation of smaller, more numerous and dynamic SGs and subsequently facilitates their clearance during the recovery period.

Across neuronal systems, NEDP1 plays pivotal roles in both neuronal development and stress adaptation. In primary rat neurons, NEDP1 functions as the major deNEDDylase acting on global neuronal substrates, with expression peaking during the first postnatal week and declining upon maturation; its activity constrains neurite outgrowth through multiple pathways, including actin cytoskeleton remodeling [141]. NEDP1-regulated NEDDylation has also emerged as a key determinant of neuronal stress responses. This pathway exhibits dynamic stress reactivity in the rodent cortex and hippocampus, where perturbations compromise neuronal viability and synaptic physiology [143]. Neuronal proteostasis is tightly linked to local translation, and under metabolic or oxidative stress, SGs assemble within dendrites and axons to regulate mRNA transport and translation [146]. Impaired SG dynamics are increasingly recognized as a contributor to neurodegeneration. Notably, NEDP1 intersects with SG turnover via conserved RNP and PARP1 pathways, where its dysregulation correlates with persistent SGs and RBP aggregation [66,147]. Evidence from tissue culture and *C. elegans* models support NEDP1 as a therapeutic target for ALS through the elimination of aberrant aggregates, and mechanistic work highlights its regulation of PARP1 as particularly significant (Figure 3) [112,119,121]. Specifically, NEDP1 inhibition promotes PARP1 hyper-NEDDylation, impairing PAR synthesis during oxidative stress. Kinetic analyses suggest this occurs at late stress stages to buffer PARP1 overactivation and prevent excess PAR production, which, while necessary for SG formation, can promote pathological SG solidification if uncontrolled [112]. This buffering function positions NEDP1 as a fine-tuner of PARP1 activity, potentially making it a more selective therapeutic target than direct PARP1 inhibition, despite the availability of potent PARP1 inhibitors. Interestingly, a recent study identified MLN4924, an inhibitor of the NEDD8-activating enzyme (NAE), as a compound that improves motor neuron viability and neuronal activity in iPSC-derived ALS models with TARDBP mutations, highlighting the therapeutic potential of targeting the NEDD8 pathway in ALS and calling for further investigation into its role in TDP-43 proteinopathies [148]. Collectively, these findings identify the NEDDylation–deNEDDylation axis as a central regulator of SG dynamics and neuronal homeostasis, with therapeutic modulation of NEDP1 shown to enhance neuronal survival and reduce pathological burden in ALS/FTD models. The proposed mechanism raises the question: Why potentially target NEDP1 over PARP1 for SG/aggregate clearance, especially since potent PARP1 inhibitors already exist?

Therapeutically, the targeting of NEDP1 offers several advantages over direct PARP1 inhibition. While PARP1 inhibitors such as Veliparib and Olaparib—widely used in cancer therapy—have shown promise in rescuing TDP-43-induced cell death in ALS models, their broad inhibition of PARP1 raises concerns due to the enzyme’s essential roles in DNA repair and transcriptional regulation [149,150]. In contrast, NEDP1 targeting reduces PARP1 activity only under stress conditions, thus preserving its physiological functions while preventing pathological hyperactivation [112]. This stress-specific modulation is supported by the non-essential nature of NEDP1 for organismal viability, as evidenced by studies in mammals and diverse model organisms including *C. elegans*, *Drosophila*, *Arabidopsis*, and *S. pombe* [94,112]. Mechanistically, NEDP1 inhibition is expected to induce a CRL-hypomorphic state by trapping the NEDD8 E2s (UBE2M/UBE2F) in auto-NEDDylated forms, while promoting the accumulation of NEDD8 chains that modulate non-cullin effectors, but loss or inhibition of NEDP1 in the studied systems does not impair survival or development [110,118,151]. However, NEDP1 knockout rather fine-tunes the cellular response to stress, particularly by modulating SG dynamics and promoting the clearance of aberrant granules.

The translational potential of NEDP1 as a therapeutic target is further underscored by the successful use of anti-NEDP1 nanobodies in ALS patient-derived fibroblasts [112,152]. These nanobodies exhibit high specificity for NEDP1, with no detectable off-target effects on the ubiquitin or other Ubl modification pathways, and effectively promote the disassembly of pathological SGs without cytotoxicity. Unlike conventional antibody therapies that target misfolded proteins directly, anti-NEDP1 nanobodies act upstream by modulating the regulatory mechanisms that control SG dynamics, offering a novel and potentially more precise strategy for treating protein aggregation disorders.

Regarding the nanobody technology itself, these molecules stand out as a promising therapeutic class due to their small size, high affinity, and low immunogenicity, which enable effective targeting, even in tissues that are traditionally difficult to access [153,154]. Although their long-term safety and efficacy require further investigation, ongoing clinical trials in cancer and autoimmune diseases highlight their growing therapeutic potential. Furthermore, Caplacizumab, the first nanobody drug approved by EMA and FDA, exemplifies successful clinical translation, paving the way for the broader use of nanobody-based therapies [155,156]. Taken together, while further research is needed, nanobodies represent an exciting frontier in biotherapeutics with the potential to transform treatment of protein aggregation and other complex disorders.

**Figure 4:**
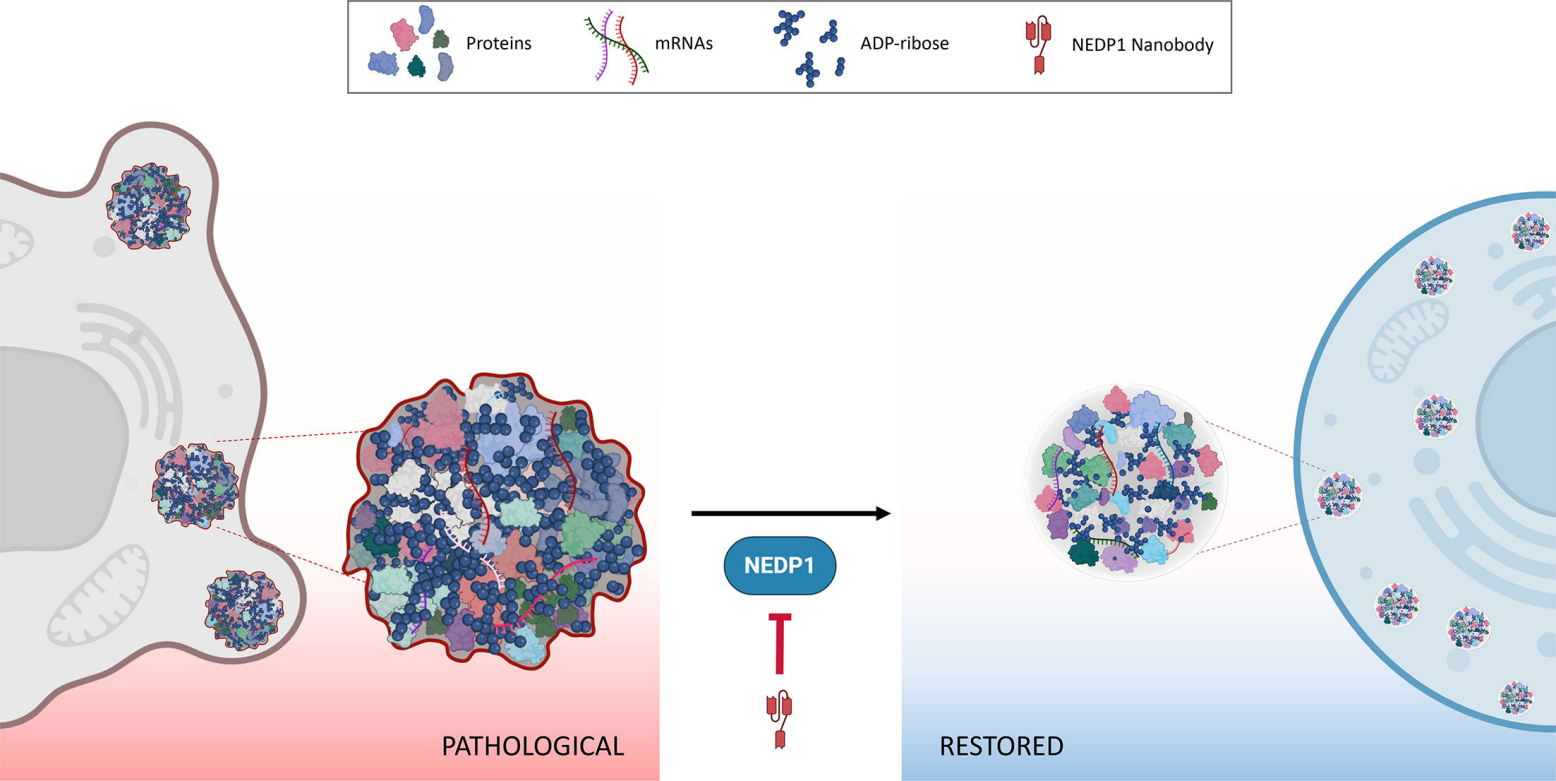
NEDP1 as a strategic modulator of SG remodeling. Inhibition of NEDP1 by nanobodies prevents pathological protein aggregation and ameliorates ALS-related phenotypes.

In summary, NEDP1 represents a component of the ubiquitin/Ubl system with promise as a potential precision therapeutic target for ALS. By modulating PARP1 activity through NEDDylation, NEDP1 inhibition offers a non-cytotoxic, stress-specific means of restoring cellular homeostasis and preventing the persistence of toxic SGs (Figure 4). Initial studies in human cell lines and *C. elegans* provide proof of principle for this strategy. Fine-tuning of the regulatory mechanisms governing SG dynamics by selectively dampening PARP1 hyperactivation avoids the pitfalls of broad-spectrum PARP1 inhibitors. This targeted control over SG remodeling and protein aggregate disassembly marks a paradigm shift—from indiscriminate inhibition to precise molecular regulation—and offers a powerful new direction for therapeutic intervention in ALS and related protein aggregation disorders. Proof of concept studies with careful evaluation of long-term effects in advanced mammalian models as well as optimizing delivery method ensuring long-term safety will be essential to evaluate the translational feasibility and clinical potential of this approach.

**Summary**

- **Significance of the field:** Protein quality control (PQC) and the regulation of stress granule (SG) dynamics are central to maintaining cellular proteostasis, especially under stress conditions that promote the accumulation of misfolded proteins. Disruptions in these processes are closely linked to the pathogenesis of neurodegenerative disorders, including amyotrophic lateral sclerosis (ALS).
- **Current insights:** SGs are cytoplasmic, membraneless organelles that transiently sequester mRNAs during cellular stress. Genetic mutations affecting SG components can lead to persistent SG-like aggregates, related to neuronal toxicity and the progression of neurodegenerative diseases.
- **Therapeutic potential:** NEDD8-specific protease 1 (NEDP1), a deconjugating enzyme that modulates NEDD8 signaling, has emerged as a pivotal regulator of SG disassembly. Its inhibition enhances SG clearance and reduces toxic protein aggregation through a mechanism dependent on poly(ADP-ribose) polymerase 1 (PARP1) activity. In disease-relevant model systems including patient-derived fibroblasts and *C. elegans* models for ALS, NEDP1 inhibition demonstrated a strong correlation between pathological aggregate clearance and amelioration of ALS-related pathological defects.
- **Outlook and future directions:** Targeting NEDP1 may present a promising and stress-specific therapeutic strategy for ALS. Importantly, this approach may circumvent the cytotoxic effects commonly associated with broad-spectrum PARP1 inhibition, offering a more tailored and safer intervention.

## Open Access

This article has been published open access under our Subscribe to Open programme, made possible through the support of our subscribing institutions, learn more here: https://portlandpress.com/pages/open_access_options_and_prices#conditional

## Abbreviations

ALS: amyotrophic lateral sclerosis
ER: endoplasmic reticulum
PQC: protein quality control
SGs: stress granules
